# Comparison of Different Image Data Augmentation Approaches

**DOI:** 10.3390/jimaging7120254

**Published:** 2021-11-27

**Authors:** Loris Nanni, Michelangelo Paci, Sheryl Brahnam, Alessandra Lumini

**Affiliations:** 1 Department of Information Engineering, University of Padua, Via Gradenigo 6, 35131 Padova, Italy; 2 BioMediTech, Faculty of Medicine and Health Technology, Tampere University, Arvo Ylpön katu 34, FI-33520 Tampere, Finland; michelangelo.paci@tuni.fi; 3 Computer Information Systems, Missouri State University, 901 S. National, Springfield, MO 65804, USA; SBrahnam@missouristate.edu; 4 Dipartimento di Informatica–Scienza e Ingegneria (DISI), Università di Bologna, Via dell’Università 50, 47521 Cesena, Italy; alessandra.lumini@unibo.it

**Keywords:** data augmentation, deep learning, convolutional neural networks, ensemble

## Abstract

Convolutional neural networks (CNNs) have gained prominence in the research literature on image classification over the last decade. One shortcoming of CNNs, however, is their lack of generalizability and tendency to overfit when presented with small training sets. Augmentation directly confronts this problem by generating new data points providing additional information. In this paper, we investigate the performance of more than ten different sets of data augmentation methods, with two novel approaches proposed here: one based on the discrete wavelet transform and the other on the constant-Q Gabor transform. Pretrained ResNet50 networks are finetuned on each augmentation method. Combinations of these networks are evaluated and compared across four benchmark data sets of images representing diverse problems and collected by instruments that capture information at different scales: a virus data set, a bark data set, a portrait dataset, and a LIGO glitches data set. Experiments demonstrate the superiority of this approach. The best ensemble proposed in this work achieves state-of-the-art (or comparable) performance across all four data sets. This result shows that varying data augmentation is a feasible way for building an ensemble of classifiers for image classification.

## 1. Introduction

Convolutional neural networks (CNNs) have revolutionized image classification. The power of these networks lies in their ability to preserve the spatial properties of images due to their highly parameterized and sparsely connected kernels. With these networks, the spatial resolution of an image is systematically downsampled, while the depth of the feature maps is simultaneously expanded. The result is a network that learns relatively low-dimensional yet powerful representations of images that, in general, greatly surpass the effectiveness of handcrafted features. The success of CNNs has led to its predominance in contemporary literature. Nearly every task domain benefiting from computer vision publishes new research reporting previously unattainable classification results using CNN as a significant component in novel systems.

With this power comes a significant disadvantage, however. The problem is that CNNs are prone to overfit on small data sets because of their massive numbers of parameters. Overfitting occurs when the network perfectly models the training set but cannot generalize its learning to predict the class of unseen data accurately. The overfitting problem has generated a need and an expectation for large data sets and is one of the pressures escalating data size growth. As noted in [1], data size is currently associated with research quality: small sample sizes are often dismissed as lacking sufficient relevancy. Unfortunately, not all domains can keep up with the new data size requirements and expectations. The availability of large data sets, for example, is problematic in medical image analysis and bioinformatics. Collecting images in these areas is well-known to be costly and labor-intensive.

Some workarounds for handling the problem of CNN overfitting include (1) transfer learning, where the network is pretrained on a massive data set (such as ImageNet [2] with its 14+ million images divided into over 1000 classes) and then finetuned for a specific problem, and (2) data augmentation, where new samples are generated that are representative of the different classes. Some other methods that reduce overfitting include dropout [3], batch normalization [3], and zero-shot/one-shot learning [4,5].

According to Shorten, et al. [6], image augmentation, the focus of this study, strikes at the heart of the problem of overfitting and aids generalizability by extracting more information from the generation of more data points, a process that fosters continuous learning. Consequently, augmentation has become a vital technology in many fields [6,7,8].

In [6], the authors divide image data augmentation into two major categories: basic image manipulations (such as flipping, transposing, and color space manipulations) and deep learning approaches (based, for example, on GANs). For reviews on the deep learning approach in data augmentation, see [9,10]; and, for some recent GAN methods specifically, see [11,12]. The aim of this study is to compare combinations of the best image manipulation methods for generating new samples that the literature has shown works well with deep learners. In Section 2, we review some of these methods. In addition, two novel image-based data augmentation algorithms are proposed: one using the Discrete Wavelet Transform (DWT) and the other the Constant-Q Gabor (CQT) transform [13]. As described in Section 3, a separate pretrained ResNet50 network is finetuned on the original training set and the new images generated by each of the augmentation algorithms. Ensembles are built from combinations of these networks and evaluated across four benchmarks: a virus data set (VIR) [14], a portrait dataset (POR) [15], a tree bark image data set (BARK) [16], and a LIGO glitches data set (GRAV) [17]. As reported in Section 4, the best ensemble proposed in this work achieves state-of-the-art (or comparable) performance across all three.

In brief, the main contributions of this study are the following:

- An evaluation across four benchmarks of some of the best augmentation methods based on image manipulations;
- The introduction of two new augmentation methods utilizing the DWT and CQT transforms (DWT achieves a top performance of 98.41% accuracy on the GRAV data set);
- An experimentally derived ensemble that achieves state-of-the-art performance on the VIR (90.00%), BARK (91.27%), POR (89.21%), and GRAV (98.33%) benchmarks. This result shows that varying data augmentation is a feasible way for building an ensemble of classifiers for image classification.
- Access to all the MATLAB source code for the experiments reported in this work (available at https://github.com/LorisNanni, accessed on 24 November 2021).

## 2. Related Works

In [6], basic image manipulations are broken down into the categories of kernel filters, color space transforms, geometric transformations, random erasing/cutting, and mixing images. These image manipulations are relatively easy to implement, but caution must be taken to preserve labels when using these transformations (flipping, for example, would change class “six” images in a written number data set to class “nine” and vice versa). Indeed, one of the most popular geometric transforms for data augmentation is flipping, especially horizontal flipping [6]. Other geometric transforms include translating and rotating an image to create new samples [18,19,20]. For augmentation purposes, rotation is best performed on the right or left axis in the range [1°, 359°] [6]. Translating by shifting up, down, left, and right focuses on different areas in the image and effectively averts positional bias in a set of images. Translation, however, often adds noise [21]. Similar in effect to translation is random cropping, which randomly samples a section of the original sample. Cropping has the additional advantage of reducing the size of the generated images if desired. Noise injection creates new images by inserting random values into them, an augmentation technique that has been explored extensively in [22]. For a comparison of geometric augmentations on AlexNet tested on ImageNet and CIFAR10 [23], see [19]; the authors in this comparison study show that rotations perform better than the other geometrical transforms discussed above.

Color often contains valuable information as witnessed by the many databases dedicated to exploring color texture: Outex [24], VisTex [25], USPtex [26], Stex [27], NewBarktex [28], KTH-TIPS 2b [29], Parquet [30] and more recently T1K+ [31]). Through color space transformations, biases in images based on illumination can be obviated [6]. For example, the pixels in the color channels of an RGB image can be put into a histogram and manipulated by applying filters to change the color space characteristics, a process that generates new samples. Color spaces can also be converted into one another for augmentation purposes, but care should be taken when transforming an RGB image into a grayscale version since this transformation has been shown to reduce performance by as much as 3%, according to [32]. Color distributions can also be jittered, and brightness, contrast, and saturation can be adjusted to make new images [18,19]. One disadvantage of using color space transformations is the risk of losing information. For a comparison between geometric and color space augmentations, see [33].

Kernel filters blur and sharpen images by sliding an n×n window across the image with a Gaussian blur or some other type of filter. A novel kernel filter called PatchShuffle that randomly swaps the matrix values in the window has also been applied with success [34].

Mixing images is another basic manipulation method that either averages pixel values between images [35] or transforms images and mixes them together in chains [36], masks, or in some other way. In [35], random images were cropped and randomly flipped horizontally. The pixel RGB channel values were then averaged to produce a new image. In [37], nonlinear methods were introduced to combine new samples. Finally, in [38], GANs were used to mix images.

Similar to random cropping, random erasing [39] and cutting [40] helps with generalizability by occluding images, beneficial since objects rarely appear in full form in the world. In [39], the authors proposed randomly erasing patches of arbitrary size in an image. This augmentation technique was evaluated on several ResNet architectures trained on CIFAR10, CIFAR100, and Fashion-MNIST, and results showed consistent performance improvements. For a survey of the literature on image mixing and data erasing, see [7].

Finally, it should be noted that some data augmentation techniques are performed considering the entire training set. Principal component analysis (PCA) jittering, for instance, multiplies the principal components of an image by a small number [18,19,33,41,42]. In [33], for instance, the first PCA component was multiplied by a random number from a uniform distribution. In [41], new samples were generated by projecting an original image onto a PCA or discrete cosine transform (DCT) subspace, adding noise to the components, and then reconstructing the altered images back into the original space.

## 3. Materials and Methods

### 3.1. Proposed Approach

**Figure 1:**
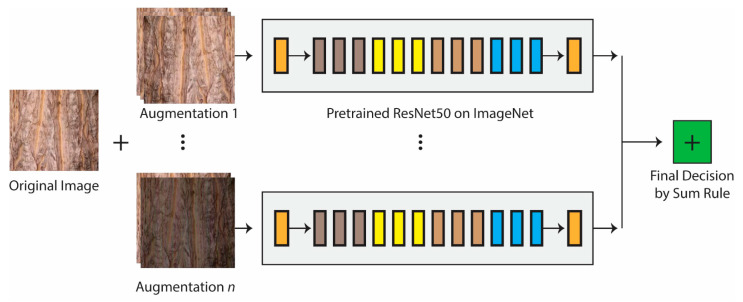
Proposed approach. Transfer learning with multiple ResNet50s pretrained on ImageNet using different sets of data augmentation methods, with networks fused by sum rule.

Consulting Figure 1, our proposed approach can be described in the following way. A given image in a training set is augmented using n augmentation methods, where n∈(0, 1, …11). The eleven augmentation methods are outlined in Section 3.2, and several combinations of these methods are experimentally investigated as described in Section 4. The original images, along with the new images generated by each augmentation method, are finetuned on separate pretrained ResNet50 [43] networks, with various combinations fused by sum rule. ResNet50 was chosen because of its low computation time to train.

**Figure 2:**
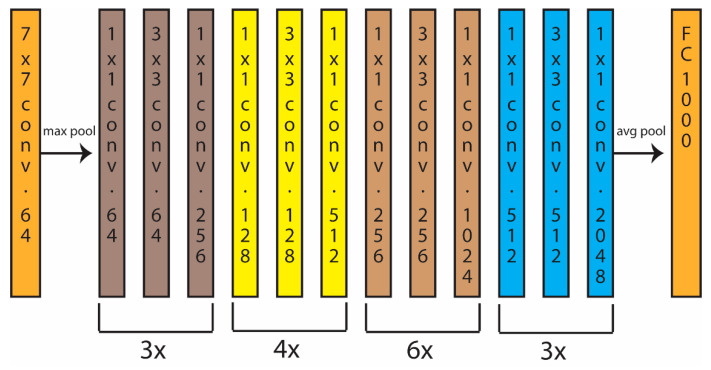
Schematic of ResNet50.

ResNet50 is a residual learning network that has 48 Convolutional layers along with 1 MaxPool and 1 Average Pool layer for a total of 50 (see Figure 2). This network can train many layers because of the addition of skip connections. In this work, each ResNet50 was pretrained on ImageNet and finetuned with a batch size of 30 and a learning rate of 0.001.

### 3.2. Data Augmentation Methods

**Figure 3:**
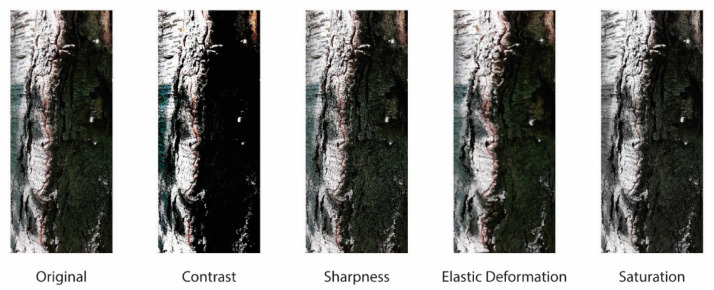
An example of some traditional augmentation methods on the BARK data set. The left image is the original image.

**Figure 4:**
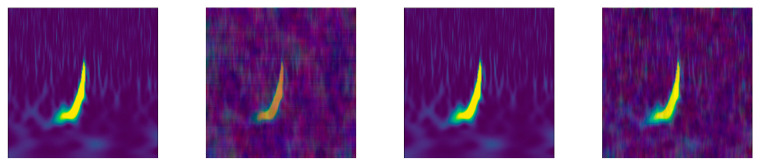
An example image of App5—DCT. The left image is the original image.

**Figure 5:**
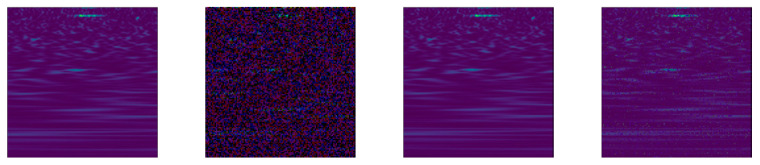
An example image of App10—DWT. The left image is the original image.

**Figure 6:**
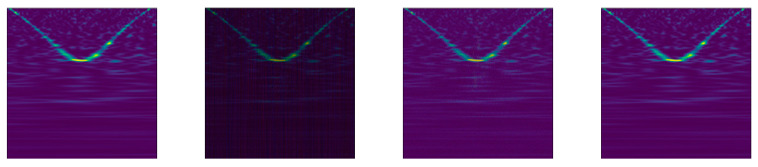
An example image of App11—DQT. The left image is the original image.

We increased the number of images in our data sets using eleven data augmentation protocols (App1–11), as detailed below. Images of some of the more traditional augmentation methods on the BARK data set are provided in Figure 3. Examples specific to App5 and the proposed methods are available in Figure 4, Figure 5 and Figure 6 using the GRAV data set.

App1. The original image is first randomly reflected in the left-right and the top-bottom directions. Subsequently, it is linearly scaled along both axes by two different factors randomly extracted from the uniform distribution [1,2].

App2. This method combines App1 with (a) image rotation, (b) translation, and (c) shear. The rotation angle is randomly extracted from [−10, 10] degrees. The translation shifts along both axes with the value randomly sampled from the interval [0, 5] pixels. The vertical and horizontal shear angles are randomly sampled from the interval [0, 30] degrees.

App3. This augmentation method is the same as App2 but without shear.

App4. This method uses PCA and is the method described in [41]. The PCA space is built on the training data only. Three perturbation methods are applied to alter the PCA coefficients representing the original image vector; these perturbations generate a new feature vector and consequently a new image after the perturbed vector is reconstructed. The first perturbation method consists of randomly setting to zero (with a probability 0.5) each element of the feature vector. In the second perturbation method, noise is added using the following MATLAB code, where PrImg is the PCA projected image:

noise = std(PrImg)/2;

K = img;

K = K + (rand(size(K))-0.5).*noise;

For the third perturbation method, five images are randomly extracted from the same class as the original image. All six images are PCA-transformed, and some of the components of the original image are exchanged with some of the corresponding components taken from the five other feature vectors. Each element of the five images replaces the original element with a probability of 0.05.

Since we have three channels for each color image, these perturbations are applied to each channel independently. In this way, App4 produces three augmented images from each original image.

App5. This augmentation method uses the same perturbation method as those described in App4, but the DCT is applied instead of PCA. The DC coefficient is never changed. Example images produced by using DCT are provided in Figure 4.

App6. This method uses contrast augmentation, sharpness augmentation, and color shifting. The contrast augmentation linearly scales the original image between two values, a and b (with a<b) provided as inputs. These two values represent the lowest and the largest intensity values in the augmented image. Every pixel in the original image with intensity less than a (or greater than b) is mapped to 0 (or 255). The sharpness augmentation first blurs the original image by a Gaussian filter with variance equal to one, and then it subtracts the blurred image from the original one. The color shifting method simply takes three integer numbers (shifts) from three RGB filters. Each shift is added to one of the three channels in the original image.

App7. This method produces seven augmented images from an original image. The first four augmented images are made by altering the pixel colors in the original image using the MATLAB function jitterColorHSV with randomly selected values for hue (in the range [0.05, 0.15]), saturation (in the range [−0.4, −0.1]), brightness (in the range [−0.3, −0.1]), and contrast (in the range [1.2, 1.4]). The fifth augmented image is simply a gaussian-filtered version of the original one generated with the MATLAB function imgaussfilt. The Gaussian filter has standard deviation randomly ranging in the range [1, 6]. The sixth augmented image is produced by the MATLAB function imsharpen with the radius of the Gaussian lowpass filter equal to one and the strength of the sharpening equal to two. A further augmented image is produced by the color shifting described in App6.

App8. This method produces two augmented images starting from the original image and a second image (the target image) randomly extracted from the same class of the original one. The two augmented images are generated using two methods based on the nonlinear mapping of the original image on the target: RGB Histogram Specification and Stain Normalization using Reinhard Method [44].

App9. This method generates six augmented images using two different methods of elastic deformation: one in-house method and an RGB adaptation of ElasticTransform from the computer vision tool Albumentations (available at https://albumentations.ai/ (accessed 15 October 2021). Both methods augment the original image by applying a displacement field to its pixels. The in-house method consists in defining, for each pixel in the original image, the displacement field Δx(x,y)=αrand(−1,+1) and Δy(x,y)=αrand(−1,+1), where α is a scaling factor that depends on the size of the original image (here 7000, 1000, and 13,000) and rand(−1,+1) represents a random value extracted from the standard uniform distribution in [−1, 1]. In the case of non-integer α values, bilinear interpolation is applied. Because of the randomness of the displacement of each pixel, this method introduces distortions in the augmented image. The second method additionally uses the displacement field Δx(x,y)=rand(−1,+1) and Δy(x,y)=rand(−1,+1) defined for each of the pixels in the original image. The horizontal Δx and the vertical Δy displacement fields are then filtered by means of one of the following three low pass filters: (1) circular averaging filter, (2) rotationally symmetric Gaussian lowpass filter, and (3) rotationally symmetric Laplacian of Gaussian filter. Finally, each of the two filtered displacement matrices is multiplied by the standard α=3000 and applied to the original image, as in the previous method (α was not optimized because it worked well with the required size of images, which is 224 × 224 for RenNet50)

App10 (NEW). To our knowledge, this augmentation approach is proposed here for the first time. It is based on DWT [45] with the Daubechies wavelet db1 with one vanishing moment. DWT produces four 114×114 matrices from the original image, containing the approximation coefficients (cA) and the horizontal, vertical, and diagonal coefficients (cH, cV and cD, respectively). Three perturbation methods are applied to the coefficient matrices. In the first method, a random number of matrix elements is set to zero for each matrix (each element with a probability of 50% is set to zero). The second method computes an additive constant as the standard deviation of the original image and a random number in the range [−0.5, 0.5]. This constant is then added to all the elements in the coefficient matrices. The third method selects five additional images from the same class as the original image and applies DWT. This process produces four coefficient matrices for each additional image. Next, each element of the original cA, cH, cV, and cD matrix is replaced (with probability 0.05) with elements from the additional image coefficient matrices. Finally, the inverse DWT is applied, generating three augmented images from the original one. Example images produced by applying this novel augmentation approach are provided in Figure 5.

App11 (NEW). To our knowledge, this augmentation method is proposed here for the first time. It is based (CQT) [13], which returns a 116×12×227 tridimensional CQT array. Like App10, three different perturbations are applied to the CQT array. The first one sets to zero a random number of elements in the CQT vector as in App10. The second perturbation computes an additive constant as the sum of the original image standard deviation and a random number in the range [−0.5, 0.5]. This constant is then added to each of the 227 bidimensional 166×12 matrices that constitute the CQT vector. Finally, the third perturbation computes the CQT of five additional images from the same class as the original image and replaces (with probability 0.05) each value in the CQT vector of the original image with CQT vector elements from the additional CQT-transformed images. Finally, the inverse CQT transform is applied, thereby producing three augmented images from the original one. Example images produced by applying this novel augmentation method are provided in Figure 6.

In Table 1, we report the number of artificial images added to each image in the original training set using the eleven approaches described above.

### 3.3. Data Sets

Benchmark data sets were selected for testing the different augmentation approaches. These data sets were chosen for the following reasons: (1) the data sets represent very different image classification problems, (2) images were collected with instruments that capture information at significantly different scales, and (3) they are publicly available and easy to access. The performance indicator for all data sets is accuracy.

In the descriptions of the data sets that follow, the names in boldface are the abbreviations used in the experimental section. These abbreviations are intended to be descriptive and reduce clutter in the tables reporting results.

VIR [14] is a popular virus benchmark containing 1500 Transmission Electron Microscopy (TEM) images (size: 41 × 41) of viruses. This data set is available at https://www.cb.uu.se/~gustaf/virustexture/ (accessed on 15 October 2021). The images in VIR are divided into fifteen classes representing different species of viruses. This virus collection contains two separate data sets: (1) the object scale data set (VIR) where the size of every virus in an image is 20 pixels and (2) the fixed scale data set where each pixel corresponds to 1 nm. Only the object scale data set is publicly available; the other is proprietary and thus not a benchmark.

BARK [16] is a relatively new data set that has reached benchmark status because it contains more than 23,000 high-resolution images (~1600 × 3800) of bark taken from twenty-three Canadian tree species, making it is the largest public data set of bark images. Bark-101 is available at http://eidolon.univ-lyon2.fr/~remi1/Bark-101/ (accessed on 15 October 2021). Each sample was collected in a region close to Quebec City and annotated by an expert. Care was taken to collect samples from trees located in different areas of the region under different illumination conditions and at widely varying scales.

GRAV [17] is another recent data set collected by the Gravity Spy project that is continuously evolving. The version used in this study is GravitySpyVersion1.0. located at https://www.zooniverse.org/projects/zooniverse/gravity-spy (accessed on 15 October 2021). The images in GRAV are related to the detection of gravitational waves via ground-based laser-interferometric detectors that are sensitive to changes smaller than the diameter of an atomic nucleus. Although these detectors are state of the art, they are still susceptible to noise, called *glitches*, that impede the search for gravitational waves. The goal of the Gravity Spy project is to detect and classify a comprehensive set of these glitches into morphological families (with such descriptive names as Power Line, Paired Doves, Scratchy, and Whistle) by combining the judgments of scientists and machine learning algorithms. GRAV contains 8583 time-frequency images (size: 470 × 570) of LIGO glitches with metadata organized into twenty-two classes. GRAV has training, validation, and testing sets to facilitate comparisons between machine learning algorithms. Four different views at different durations can be extracted from each glitch.

POR [15] is a data set that contains 927 paintings from six different art movements: (1) High Renaissance, (2) Impressionism, (3) Northern Renaissance, (4) Post-Impressionism, (5) Rococo, and (6) Ukiyo-e. The authors of this data set report a best accuracy rate of 90.08% using a ten-fold cross-validation protocol and a method that combines both deep learning and handcrafted features.

## 4. Experimental Results

In the experiments reported in Table 2, we compare the results of ResNet50 coupled with different data augmentation approaches. We also report the performance of the following ensembles:

1. EnsDA_all: this is the fusion by sum rule among all the ResNet50 trained using all eleven data augmentation approaches; a separate ResNet50 is trained for each of the data augmentation approaches. The virus data set has gray level images; for this reason, the three data augmentation methods based on color (App6–8) perform poorly on VIR, so these methods are not used for VIR.
2. EnsDA_5: this is a fusion where only five ResNet50 networks are trained, a separate one on the first five data augmentation approaches (App1–5).
3. EnsBase: this is a baseline approach intended to validate the performance of EnsDA_all; EnsBase is an ensemble (combined by sum rule) of eleven ResNet50 networks each trained only on App3, selected because it obtains the highest average performance among all the data augmentation approaches.
4. EnsBase_5: this is another baseline approach intended to validate the performance of EnsDA_5; it is an ensemble of five ResNet50 with each coupled with App3.

The first row of Table 2 (NoDA), reports performance obtained by a ResNet50 without data augmentation. The last row of Table 2 (State of the art) reports the best performance reported in the literature on each of the data sets: VIR [46], BARK [47], GRAV [17], and POR [15]). In [46], which reports the best performance on VIR, features were extracted from the deeper layers of three pretrained CNNs (Densenet201, ResNet50, and GoogleNet), transformed into a deep co-occurrence representation [48] and trained on separate SVMs that were finally fused by sum rule. As the deeper layers of a CNN produce high-dimensional features, dimensionality reduction was performed using DCT [49]. In [47], which obtains the best performance on the BARK data set, a method based on 2D spiral Markovian texture features (2DSCAR) via multivariate Gaussian distribution was trained on a 1-NN with Jeffery’s divergence as the distance measure. In [47], which provides the best performance on GRAV, several ensembles were built from extracted views using a set of basic classifiers that included an SVM and two merge-view models proposed in [50]. The best performing ensemble in that study was fused by weighted sum rule. In [15], the authors obtain 80.09% on POR using their deep learning approach (the focus here) and 90.08% when combining handcrafted with deep learning features. For fair comparison, the 80.09% on the deep learners should be compared with our method.

Examining Table 2, the following conclusions can be drawn:

- Data augmentation approaches strongly boost performance, as evident by comparing the ensembles using augmentation to the low performance of NoDA (well known in the literature).
- There is no clear winner among the data augmentation approaches; in each data set, the best method is different.
- The best performance is obtained by EnsDA_all; this ensemble obtains the best performance, even when compared with the state of the art, on all the data sets. This result shows that varying data augmentation is a feasible way for building an ensemble of classifiers for image classification.
- Refs. [29,33], two previous methods for data augmentation based on PCA, clearly works poorly compared with our PCA-based approach.

Finally, in Table 3 and Table 4, we compare EnsDA_all with the best reported in the literature for VIR and BARK. As can be observed, our proposed method obtains state-of-the-art performance.

In [17], the best reported performance by the ensemble proposed in that paper was 98.21%, lower than our 98.33%.

## 5. Discussion

The goal of this study was to compare combinations of the best image manipulation methods for generating new image data points. Original images and sets of many augmented images were trained, each on a separate ResNet50 network. In addition, two new augmentation methods were proposed: one based on the DWT and the other on the CQT transform. These networks were compared, combined, and evaluated across four benchmarks representing diverse image recognition tasks. The best ensemble proposed in this work achieved state-of-the-art performance across all four benchmarks, with the new data augmentation method based on DWT alone achieving top performance on one of the data sets.

This study demonstrates the power of combining data augmentation for increasing CNN performance. The method developed in this paper should perform well on many image classification problems. However, we recognize that the results reported here use only a few image manipulation methods for data augmentation and were tested on only four data sets. Based on the results reported in this study, our plans for the future include testing more sets of data augmentation approaches, including those based on deep learners, such as GANS, across many more data sets.

## Figures and Tables

**Table 1:** Number of artificial images created by each data augmentation method.

Data Augmentation Method	Number of Generated Images
App1	3
App2	6
App3	4
App4	3
App5	3
App6	3
App7	7
App8	2
App9	6
App10	3
App11	3

Note: The number of generated images is per image in the training set. As an example, if a training set has 1000 images, then App1 would build an additional 3 × 1000 images. Thus, the final training set would be 1000 (the original number in the training set) plus the 3000 images generated by App1.

**Table 2:** Performance (accuracy) of the different configurations for data augmentation.

DataAUG	VIR	BARK	GRAV	POR
NoDA	85.53	87.48	97.66	86.29
App1	87.00	89.60	97.83	87.05
App2	86.87	90.17	98.08	85.97
App3	87.80	89.45	97.99	87.05
App4	86.33	87.91	97.74	84.90
App5	86.00	87.61	97.83	86.41
App6	--	88.63	98.08	87.37
App7	--	89.28	97.99	88.13
App8	--	87.29	97.74	86.06
App9	85.67	88.86	98.24	86.19
App10	84.20	86.39	98.41	85.10
App11	85.47	89.20	97.91	86.71
[29]	82.93	--	--	--
[33]	83.07	--	--	--
EnsDA_all	90.00	91.27	98.33	89.21
EnsDA_5	89.60	91.01	98.08	88.56
EnsBase	89.73	90.67	98.16	87.58
EnsBase_5	89.60	90.66	97.99	87.48
State of the art	89.60	90.40	98.21	80.09/90.08 *

* As noted above, for fair comparison, 80.09 is the best performance using their deep learning approach, but 90.08 was obtained when combining handcrafted with deep learning features. Note: the virus data set has gray level images; for this reason, the three data augmentation methods based on color (App7–8) perform poorly on VIR, so these methods are not reported for this data set. Additionally, because of the low performance on VIR, [29,33] are not tested on BARK, GRAV, and POR. Bold values highlight the best results.

**Table 3:** Performance (accuracy) compared with the best in the literature on the VIR data set.

EnsDA_all	[46]	[51]	[52]	[53]	[54]	[14]	[53]	[55]
90.00	89.60	89.47	89.00	88.00	87.27	87.00 *	86.20	85.70

Note: the method notated with * combines descriptors based on both object scale and fixed scale images (as noted in Section 3.3, the fixed scale data set is not publicly available); yet, even with this advantage, our proposed system outperforms [14].

**Table 4:** Comparison with the literature, BARK data set.

EnsDA_all	[56]	[57]	[47]	[16]
91.27	48.90	85.00	90.40	85.00

## Data Availability

Publicly available data sets were analyzed in this study. The MATLAB code for all the data augmentation methods is available at https://github.com/LorisNanni (accessed on 24 November 2021).
